# Supplementary material for: Global distribution and conservation status of ecologically rare mammal and bird species
Source: Nat Commun. 2020 Oct 8;11:5071. doi: 10.1038/s41467-020-18779-w (PMC7545165; doi:10.1038/s41467-020-18779-w)
Supplement: Supplementary file 1 — Supplementary Information [file 41467_2020_18779_MOESM1_ESM.pdf]

SUPPLEMENTARY MATERIALS

Global distribution and conservation status of ecologically rare mammal and bird species

Loiseau & Mouquet et al.

This PDF file includes:

Supplementary Tables 1 to 3

Supplementary Figures 1 to 15

Global biodiversity scenarios supplementary methods

Supplementary Tables

Supplementary Table 1: Correlations between functional traits and the first four axes of the PcoA of mammals (explaining respectively 29.3%, 21.8%, 13.5%, 8.2% of the total variance). For numerical traits we used Spearman correlations and for nominal traits we used Goodman and Kruskal's gamma correlations. The first axis of functional differentiation integrated both a body mass gradient (R^2 = *- 0.58*) and diet (herbivores R^2 = *-0.80* vs. invertebrate-eaters R^2 = *0.68*). The second axis integrated both, body mass gradient (body mass, R^2 = *-0.45*) and diet (frugivores R^2 = *-0.61* vs. invertebrate-eaters R^2 = *0.53*). The third axis integrated mostly diet (granivores R^2 = *-0.26* vs. invertebrate-eaters R^2 = *0.40*). The fourth axis integrated mostly diet (scavenger R^2 = *-0.41* vs. herbivores R^2 = *0.44*).

Functions	Traits	Correlation	PC1	PC2	PC3	PC4
Diet	Invertebrate	Spearman	0.68	0.53	0.40	-0.07
Diet	Mammals/birds	Spearman	0.01	0.06	0.01	-0.18
Diet	Reptiles	Spearman	0.11	0.28	-0.005	-0.32
Diet	Fish	Spearman	-0.02	0.10	0.06	-0.10
Diet	Vertebrates	Spearman	0.01	-0.08	0.08	-0.15
Diet	Scavenge	Spearman	0.11	0.41	0.02	-0.41
Diet	Fruit	Spearman	0.01	-0.62	-0.22	-0.36
Diet	Nectar	Spearman	0.17	-0.23	-0.05	-0.10
Diet	Seed	Spearman	-0.24	-0.06	-0.26	-0.15
Diet	Plant	Spearman	-0.80	-0.23	-0.23	0.44
Foraging stratum	Foraging stratum	Kruskal	0.34	0.38	0.1	0.24
Activity	Nocturnal	Kruskal	0.15	0.13	0.25	0.02
Activity	Crepuscular	Kruskal	0.21	0.01	0.11	0.02
Activity	Diurnal	Kruskal	0.26	0.03	0.33	0.01
BodyMass	BodyMass	Spearman	-0.60	-0.45	-0.09	0.01

Supplementary Table 2: Correlations between functional traits and the first four axes of the PcoA of birds (explaining respectively 26.4%, 17.8%, 11.4%, 8.6% of the total variance). For numerical traits we used Spearman correlations and for nominal traits we used Goodman and Kruskal's gamma correlations. The first axis of functional differentiation integrated mostly foraging strata position (ground $R^2 = -0.88$ vs. middle high $R^2 = 0.82$). The second axis integrated mostly diet (invertebrate-eaters $R^2 = -0.90$ vs. frugivores $R^2 = 0.50$). The third axis integrated mostly foraging strata position (forest understory $R^2 = -0.84$ vs. canopy $R^2 = 0.39$). The fourth axis integrated foraging strata position (water surface $R^2 = 0.37$ vs. ground $R^2 = -0.29$) and diet (piscivores $R^2 = 0.36$ vs. frugivores $R^2 = -0.62$).

Functions	Traits	Correlation	PC1	PC2	PC3	PC4
Diet	Invertebrates	Spearman	-0.12	-0.91	0.21	-0.03
Diet	Mammals/birds	Spearman	-0.15	0.14	0.10	0.18
Diet	Reptiles	Spearman	-0.17	0.05	0.13	0.11
Diet	Fish	Spearman	-0.14	0.11	0.30	0.36
Diet	Vertebrates	Spearman	-0.05	0.02	0.04	0.03
Diet	Scavenge	Spearman	-0.13	0.11	0.08	0.10
Diet	Fruit	Spearman	0.40	0.51	-0.003	-0.62
Diet	Nectar	Spearman	0.36	0.11	-0.22	0.23
Diet	Seed	Spearman	-0.34	0.42	-0.30	-0.17
Diet	Plant	Spearman	-0.15	0.33	-0.10	0.03
Foraging stratum	below the water surfaces	Spearman	-0.07	0.09	0.23	0.32
Foraging stratum	<5 inches below water surface	Spearman	-0.20	0.09	0.30	0.38
Foraging stratum	ground	Spearman	-0.88	0.16	-0.03	-0.29
Foraging stratum	understory	Spearman	0.21	-0.40	-0.84	0.15
Foraging stratum	Middle high tree	Spearman	0.82	-0.03	0.02	-0.16
Foraging stratum	above tree canopy	Spearman	0.61	0.21	0.39	-0.16
Foraging stratum	ForStrat-aerial	Spearman	0.08	-0.04	0.1	0.19
Diet	predominantly pelagic	Kruskal	0.002	0.01	0.05	0.07
Nocturnal	foraging activity at night	Kruskal	0.004	0.0001	0.004	0.04

Bodymass	body mass	Spearman	-0.28	0.45	0.34	0.02

Supplementary Table 3: P-values indicate if there is or not a significant difference between ecologically rare, common, and average species via one-way ANOVA and Tukey's post-hoc tests. From left to right: Human Footprint that measure the cumulative impact of direct human pressures; Human development index (HDI); Change in distribution range based on climate change projections (scenario RCP 8.5, Horizon 2041-2060 and 2061-2080) and Target achievement (extent to which species are represented within PAs regarding their restrictiveness). P-value in bold are significant ($P < 0.05$).

Pairs	Human Footprint	HDI	Conflicts	Climate Change 2041-2060	Climate Change 2061-2080	Target Achievement
Mammals						
Common - Average	**6.9e-03**	0.25	0.88	0.32	0.37	**< 1.e-16**
Rare - Average	**8.3e-05**	**4.2e-09**	0.09	**0.01**	0.09	0.27
Rare - Common	**9.7e-08**	**3.2e-03**	0.47	**2.4e-03**	**0.02**	**< 1.e-16**
Birds						
Common - Average	**3.4e-04**	**2.7e-06**	**7.2e-04**	0.07	0.07	**9.2e-11**
Rare - Average	**3.5e-09**	**1.7e-08**	**3.3e-05**	**0.01**	**6.2e-03**	0.06
Rare - Common	**3.5e-09**	0.72	0.8	**3.5e-04**	**1.2e-04**	**9.2e-11**

Supplementary Figures

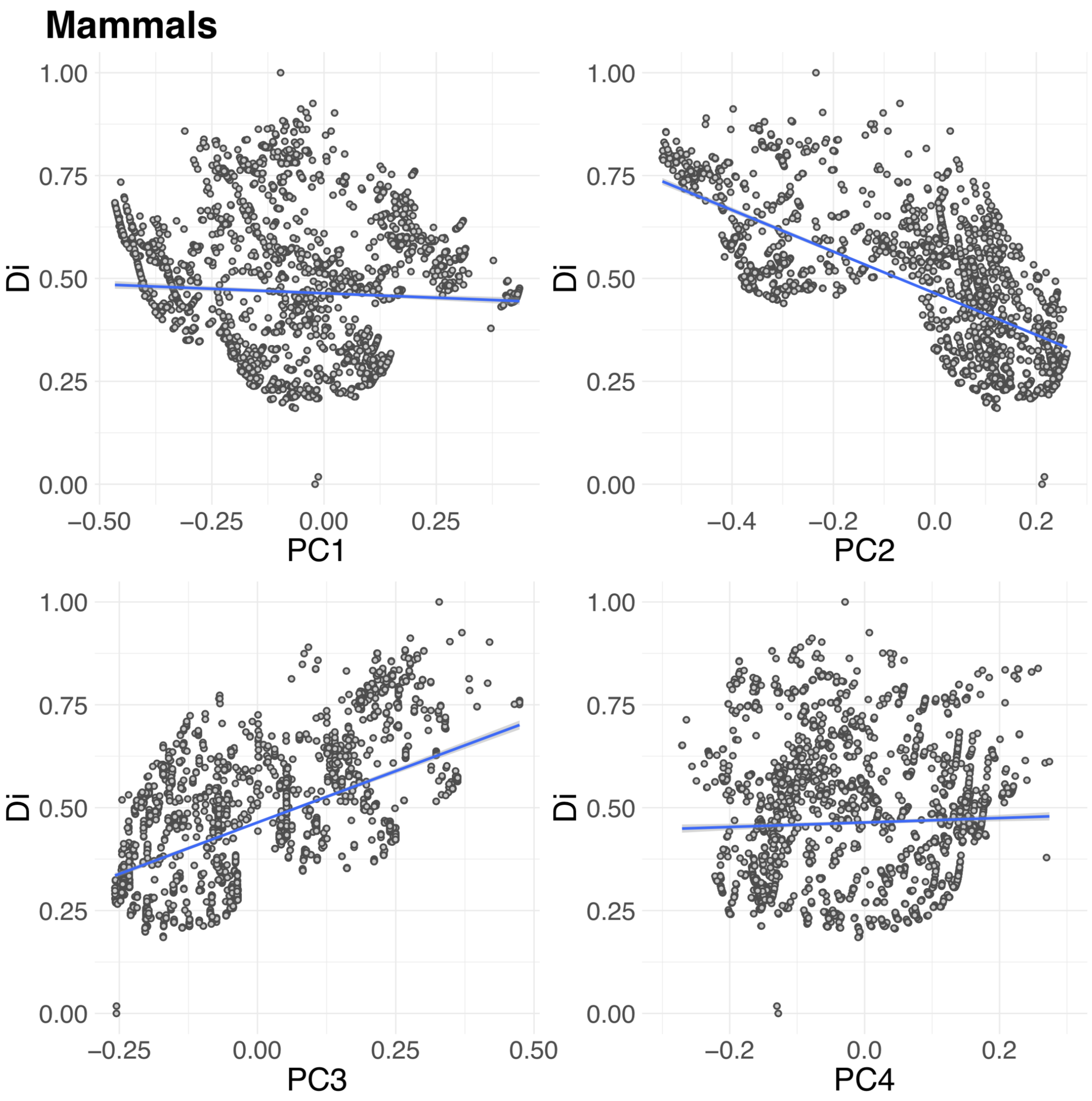

Supplementary Figure 1: Relation between functional distinctiveness (Di) and the four axes of the functional traits PcoA for mammals. Blue line represents the linear model between Di and PcoA axes. Distinctiveness was mainly correlated with axes 2 and 3 of the PcoA ($R^2 = 0.51$, $R^2 = 0.31$, *p-values* < 0.001. Note that to limit effect of heteroscedacity, we perform a Box-cox transformation, a mathematical transformation of the variable to make it approximate to a normal distribution and test linear model.

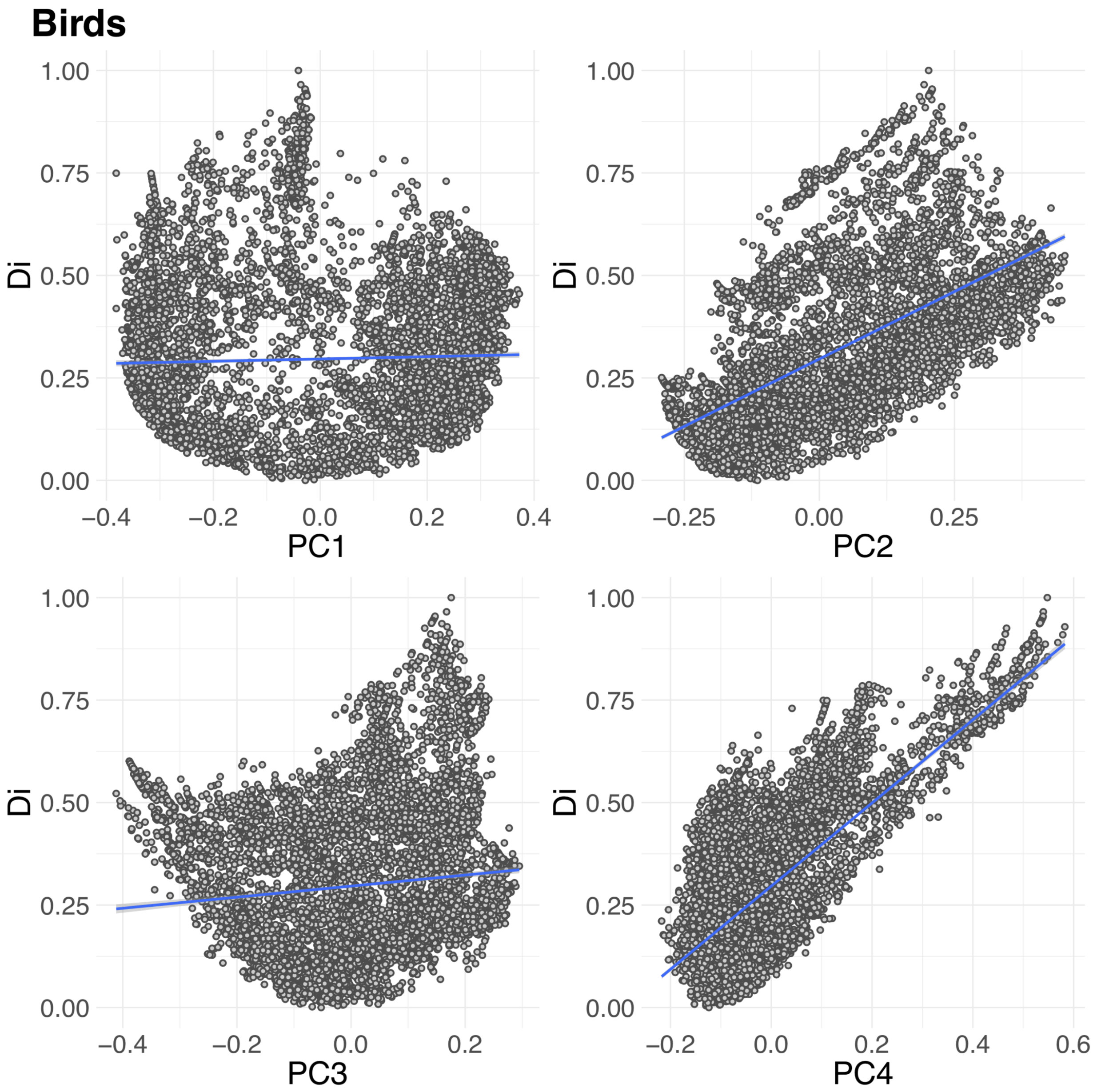

Supplementary Figure 2: Relation between functional distinctiveness (Di) and the four axes of the functional traits PcoA for birds. Blue line represents the linear model between Di and PcoA axes. Distinctiveness was mainly correlated with axes 2 and 4 of the PcoA ($R^2 = 0.42$, $R^2 = 0.48$, *p-values* < 0.001. Note that to limit effect of heteroscedacity, we perform a Box-cox transformation, a mathematical transformation of the variable to make it approximate to a normal distribution and test linear model.

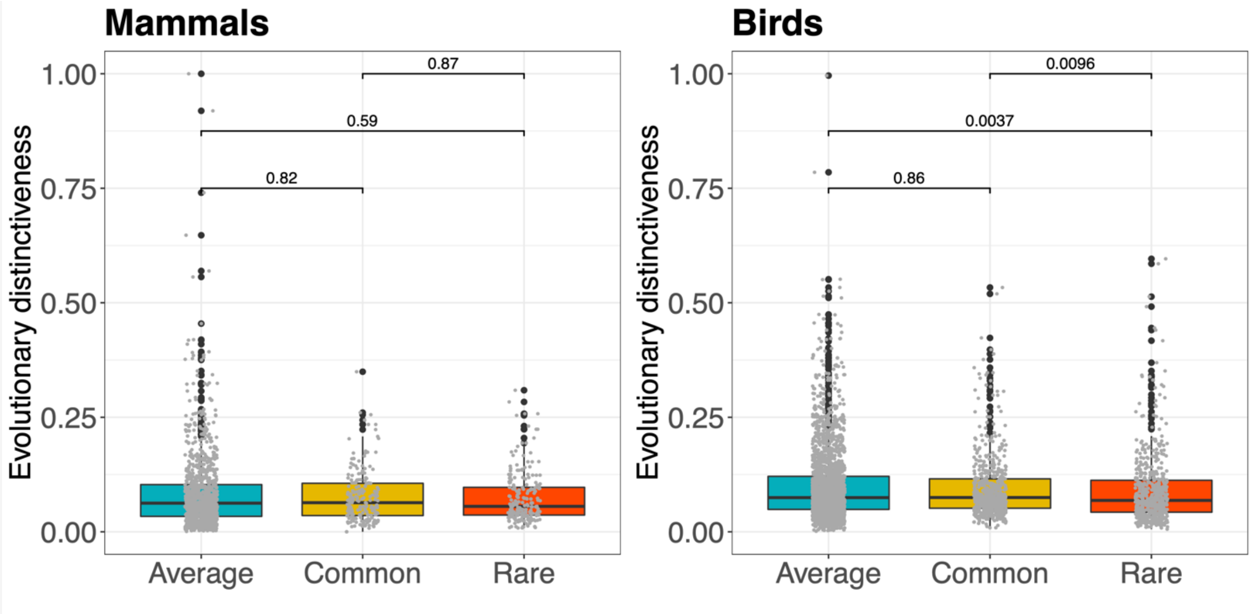

Supplementary Figure 3: Evolutionary distinctiveness of ecologically common mammals (left) and birds (right) (blue, n = 1126 & n = 2417), average (orange, n = 200 & n = 569) and rare (red, n = 237 & n = 573). The Evolutionary Distinctiveness of species i, is high when the species has a long unshared branch length with all the other species. The more "isolated" a species is in a phylogenetic tree, the higher its evolutionary distinctiveness. First line of the boxplot represents the first quartile (25th Percentile) of the distribution, bold line represents median, third line represents the third quartile (5th Percentile). The two whisker boundaries represent 1.5 time the interquartile space. The difference was tested with Wilcox test. Significant P-values between rare and common and rare and average bird species, despite no clear patterns can be explain by the statistical power due to the high number of samples.

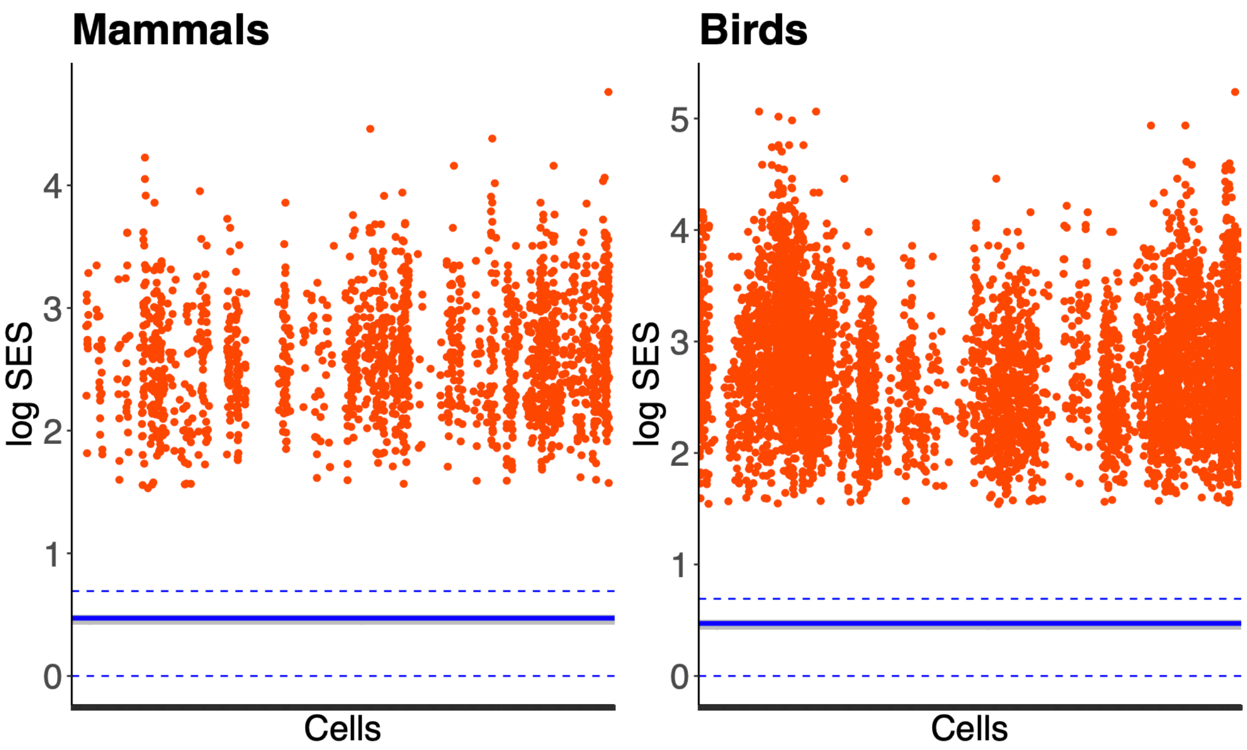

Supplementary Figure 4: Standardized effect size (SES) of the number of species on the number of ecologically rare species per cells. For clarity we plotted SES on a log scale log(SES + abs(min(SES))+1). Solid blue line indicate SES = 0 (0.47), for random values. Bluedotted lines indicate the α = 0.05 threshold of SES = 1.96 (0-0.69) for significantly non-random values. Each data point represents a cell. Values above the null expectation indicate that the cell host more ecologically rare species than expected given the number of species in the cell. In red, cells hosting at least on ecologically rare species, in grey, cells that do not host any ecologically rare species.

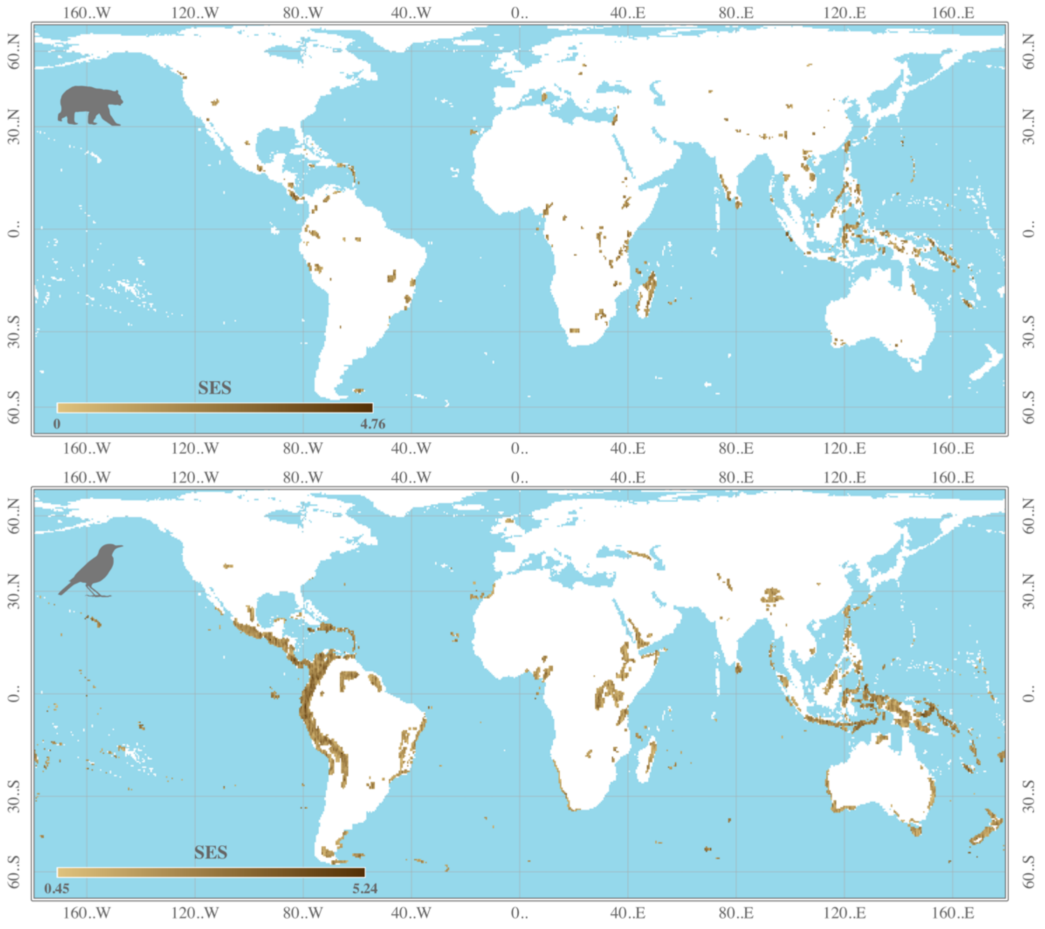

Supplementary Figure 5 Global distribution considering only unglaciated areas of Standardized effect size (SES) of the number of species on the number of ecologically rare species per cells for mammals (top) and birds (bottom) species. Cells in brown host more ecologically rare species. For clarity we plotted SES on a log scale log(SES + abs(min(SES))+1).

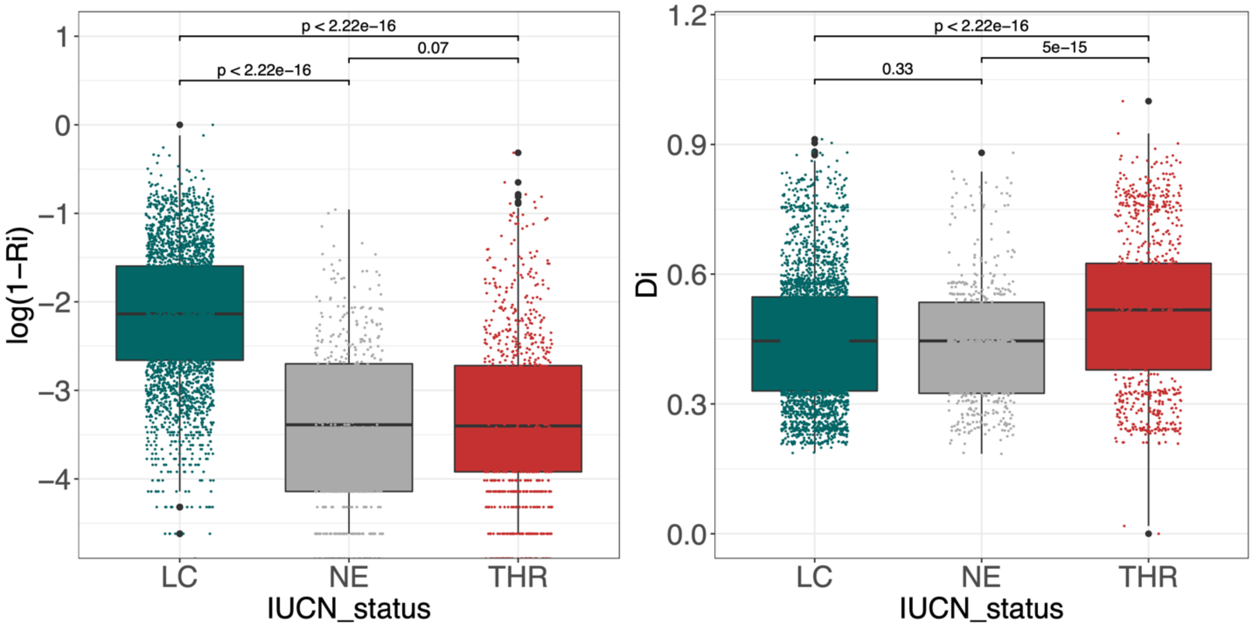

Supplementary Figure 6: Mammals geographical restrictiveness (Ri, we use log(1-Ri) for clarity as most of the Ri values are close to 0 for mammals and birds) and distinctiveness (Di) in relation to IUCN status according to criteria by the IUCN Red List, Threatened (TH red: Vulnerable, Endangered, Critically Endangered, n = 978), Least concern (LC green: Least Concern and Near Threatened, n = 3140) and None Evaluated (NE grey: Not Evaluated or inadequate information or data deficient, n = 536). Each point represents a species. The difference between IUCN Status was tested with Wilcox test and is shown along with the p-value of this difference. First line of the boxplot represents the first quartile (25th Percentile) of the distribution, bold line represents median, third line represents the third quartile (5th Percentile). The two whisker boundaries represent 1.5 time the interquartile space.

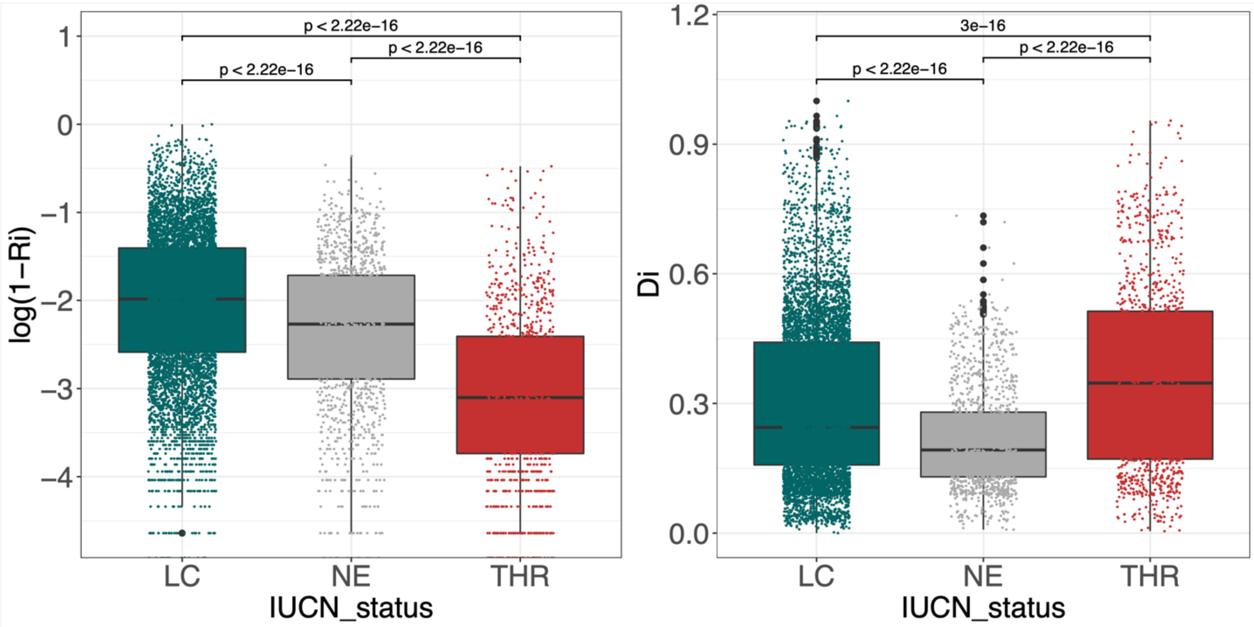

Supplementary Figure 7: Birds geographical restrictiveness (Ri, we use log(1-Ri) for clarity as most of the Ri values are close to 0 for mammals and birds) and distinctiveness (Di) in relation to IUCN status according to criteria by the IUCN Red List, Threatened (TH red: Vulnerable, Endangered, Critically Endangered, n = 1081), Least concern (LC green: Least Concern and Near Threatened, n = 6875) and None Evaluated (NE grey: Not Evaluated or inadequate information or data deficient, n = 1249). Each point represents a species. The difference between IUCN Status was tested with Wilcox test and is shown along with the p-value of this difference. First line of the boxplot represents the first quartile (25th Percentile) of the distribution, bold line represents median, third line represents the third quartile (5th Percentile). The two whisker boundaries represent 1.5 time the interquartile space.

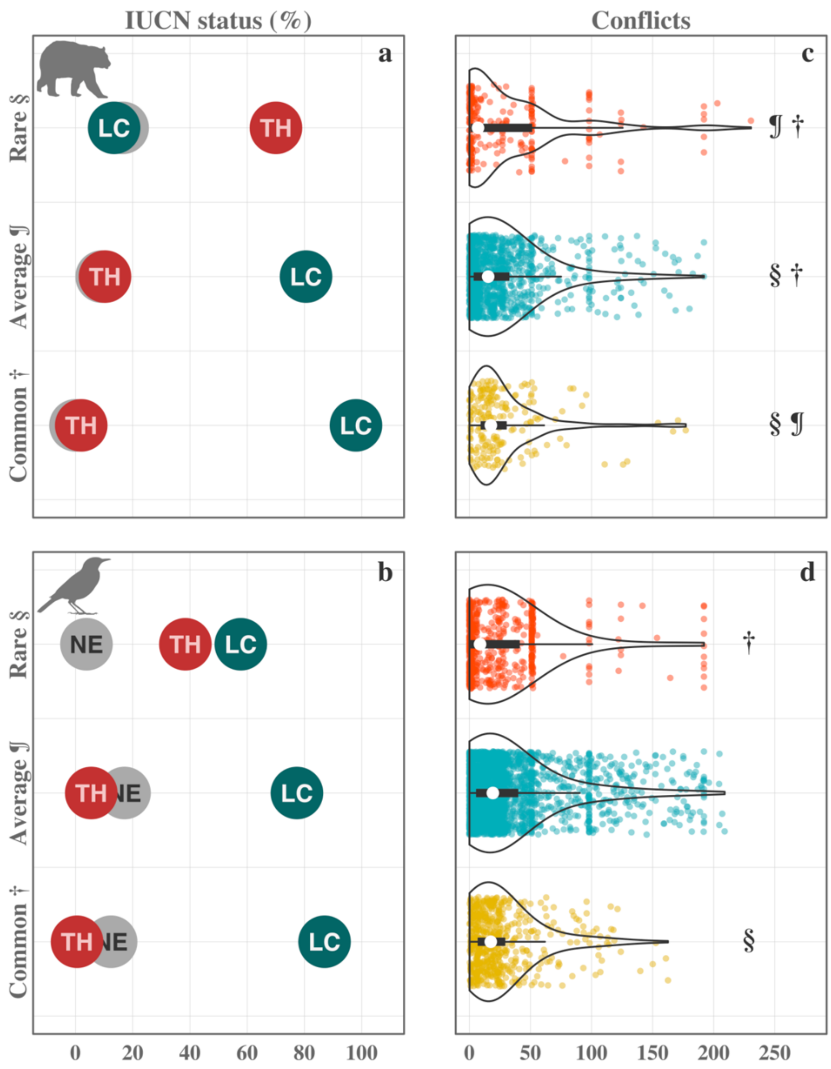

Supplementary Figure 8: Number of conflicts on country hosting ecologically rare (red), average (blue) and common (orange) species, Letters indicate significant similar distribution between groups (*$P < 0.05$*, See Supplementary Table 3 for all p-value) via one-way ANOVA and Tukey's post-hoc tests.

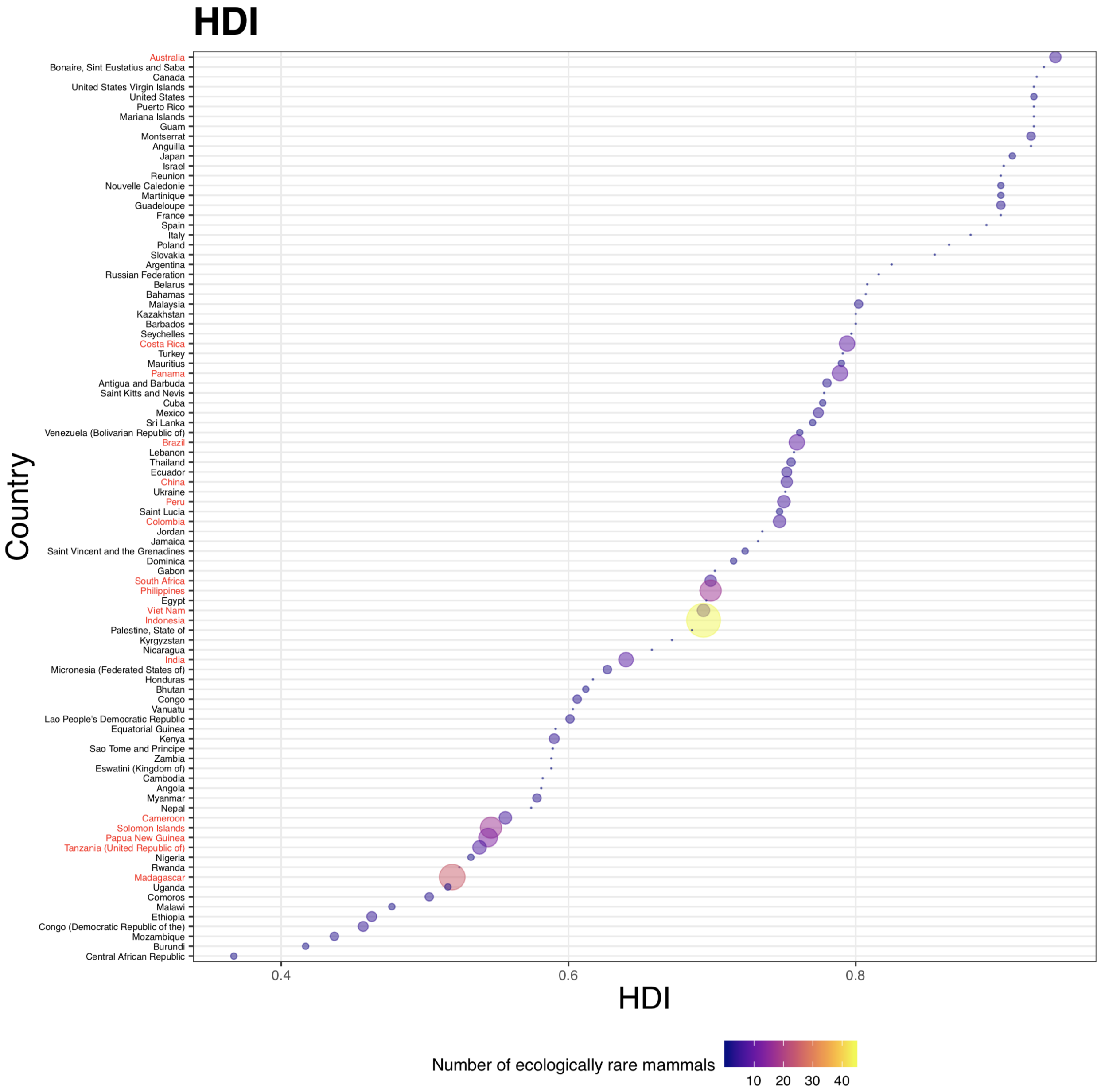
HDI
Australia
Bonaire, Sint Eustatius and Saba
Canada
United States Virgin Islands
United States
Puerto Rico
Mariana Islands
Guam
Montserrat
Anguilla
Japan
Israel
Reunion
Nouvelle Caledonie
Martinique
Guadeloupe
France
Spain
Italy
Poland
Slovakia
Argentina
Russian Federation
Belarus
Bahamas
Malaysia
Kazakhstan
Barbados
Seychelles
Costa Rica
Turkey
Mauritius
Panama
Antigua and Barbuda
Saint Kitts and Nevis
Cuba
Mexico
Sri Lanka
Venezuela (Bolivarian Republic of)
Brazil
Lebanon
Thailand
Ecuador
China
Ukraine
Peru
Saint Lucia
Colombia
Jordan
Jamaica
Saint Vincent and the Grenadines
Dominica
Gabon
South Africa
Philippines
Egypt
Viet Nam
Indonesia
Palestine, State of
Kyrgyzstan
Nicaragua
India
Micronesia (Federated States of)
Honduras
Bhutan
Congo
Vanuatu
Lao People's Democratic Republic
Equatorial Guinea
Kenya
Sao Tome and Principe
Zambia
Eswatini (Kingdom of)
Cambodia
Angola
Myanmar
Nepal
Cameroon
Solomon Islands
Papua New Guinea
Tanzania (United Republic of)
Nigeria
Rwanda
Madagascar
Uganda
Comoros
Malawi
Ethiopia
Congo (Democratic Republic of the)
Mozambique
Burundi
Central African Republic
Country
0.4
0.6
0.8
HDI
Number of ecologically rare mammals
10
20
30
40

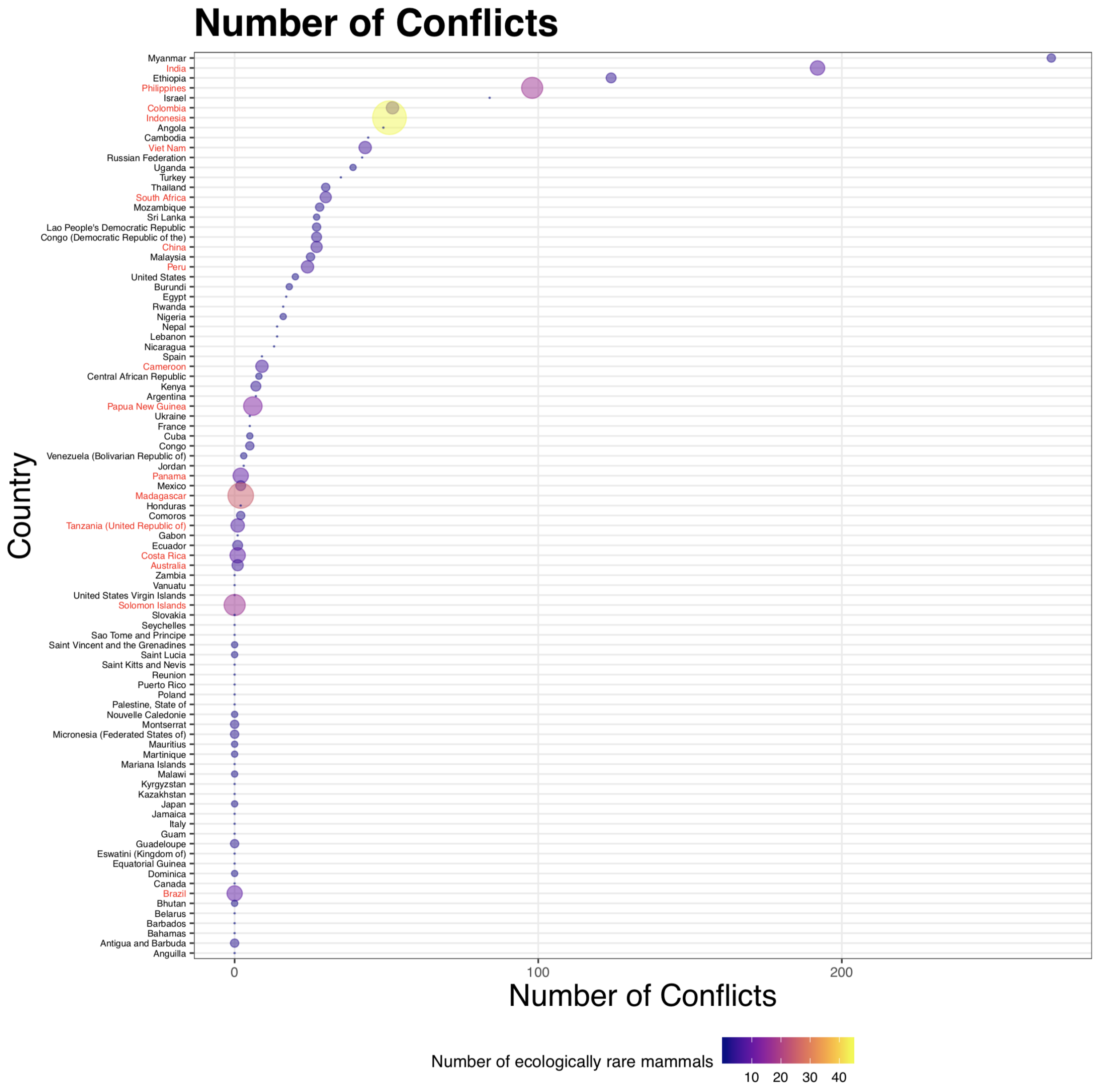

Supplementary Figure 9: HDI (top) and number of conflicts (bottom) in countries hosting ecologically rare mammals (92 countries). In red we highlight countries with more than 5 ecologically rare species.

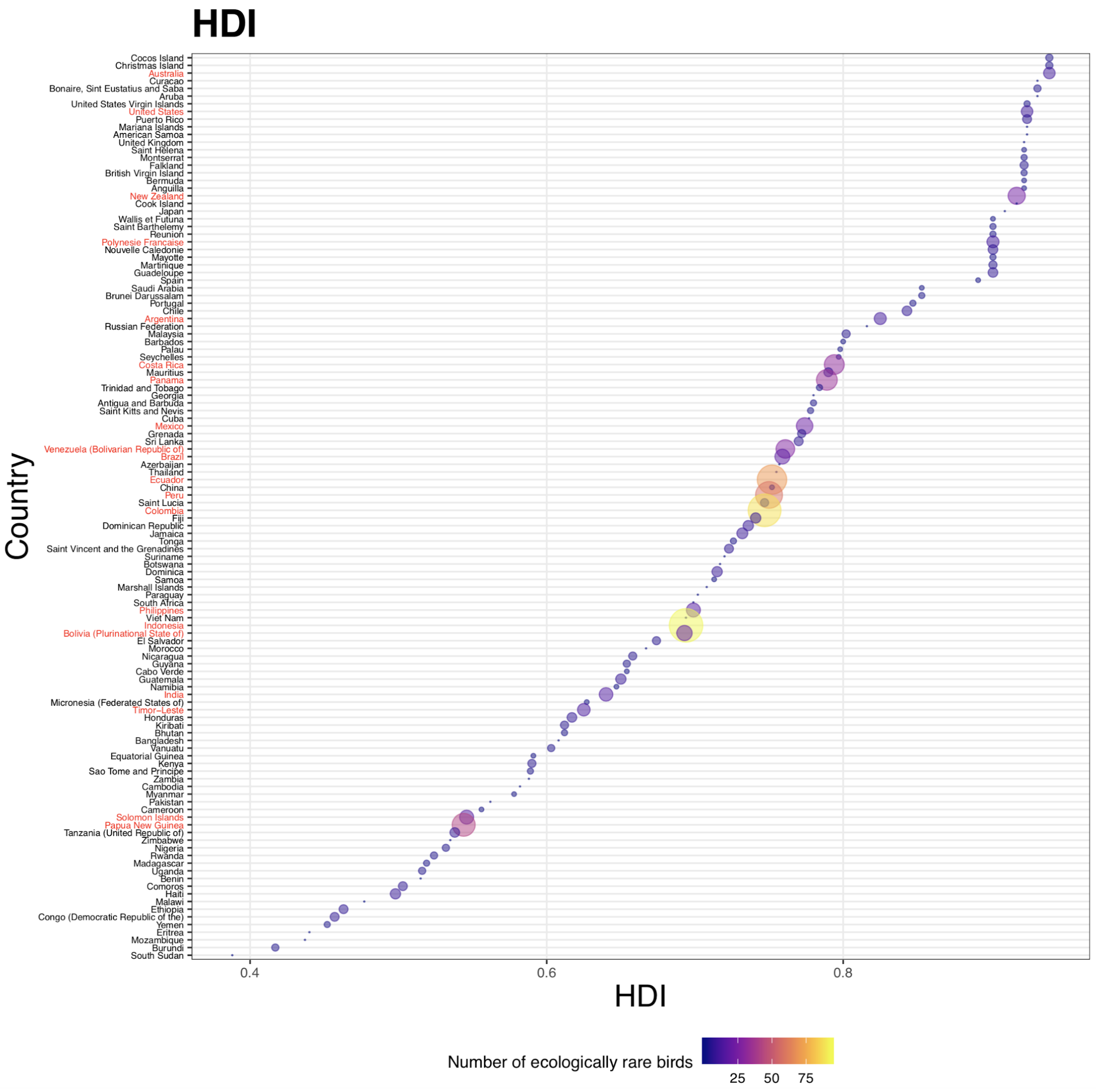

HDI
Country
Cocos Island
Christmas Island
Australia
Curacao
Bonaire, Sint Eustatius and Saba
Aruba
United States Virgin Islands
United States
Puerto Rico
Mariana Islands
American Samoa
United Kingdom
Saint Helena
Montserrat
Falkland
British Virgin Island
Bermuda
Anguilla
New Zealand
Cook Island
Japan
Wallis et Futuna
Saint Barthelemy
Reunion
Polynesie Francaise
Nouvelle Caledonie
Mayotte
Martinique
Guadeloupe
Spain
Saudi Arabia
Brunei Darussalam
Portugal
Chile
Argentina
Russian Federation
Malaysia
Barbados
Palau
Seychelles
Costa Rica
Mauritius
Panama
Trinidad and Tobago
Georgia
Antigua and Barbuda
Saint Kitts and Nevis
Cuba
Mexico
Grenada
Sri Lanka
Venezuela (Bolivarian Republic of)
Brazil
Azerbaijan
Thailand
Ecuador
China
Peru
Saint Lucia
Colombia
Fiji
Dominican Republic
Jamaica
Tonga
Saint Vincent and the Grenadines
Suriname
Botswana
Dominica
Samoa
Marshall Islands
Paraguay
South Africa
Philippines
Viet Nam
Indonesia
Bolivia (Plurinational State of)
El Salvador
Morocco
Nicaragua
Guyana
Cabo Verde
Guatemala
Namibia
India
Micronesia (Federated States of)
Timor−Leste
Honduras
Kiribati
Bhutan
Bangladesh
Vanuatu
Equatorial Guinea
Kenya
Sao Tome and Principe
Zambia
Cambodia
Myanmar
Pakistan
Cameroon
Solomon Islands
Papua New Guinea
Tanzania (United Republic of)
Zimbabwe
Nigeria
Rwanda
Madagascar
Uganda
Benin
Comoros
Haiti
Malawi
Ethiopia
Congo (Democratic Republic of the)
Yemen
Eritrea
Mozambique
Burundi
South Sudan
0.4
0.6
0.8
HDI
Number of ecologically rare birds
25
50
75

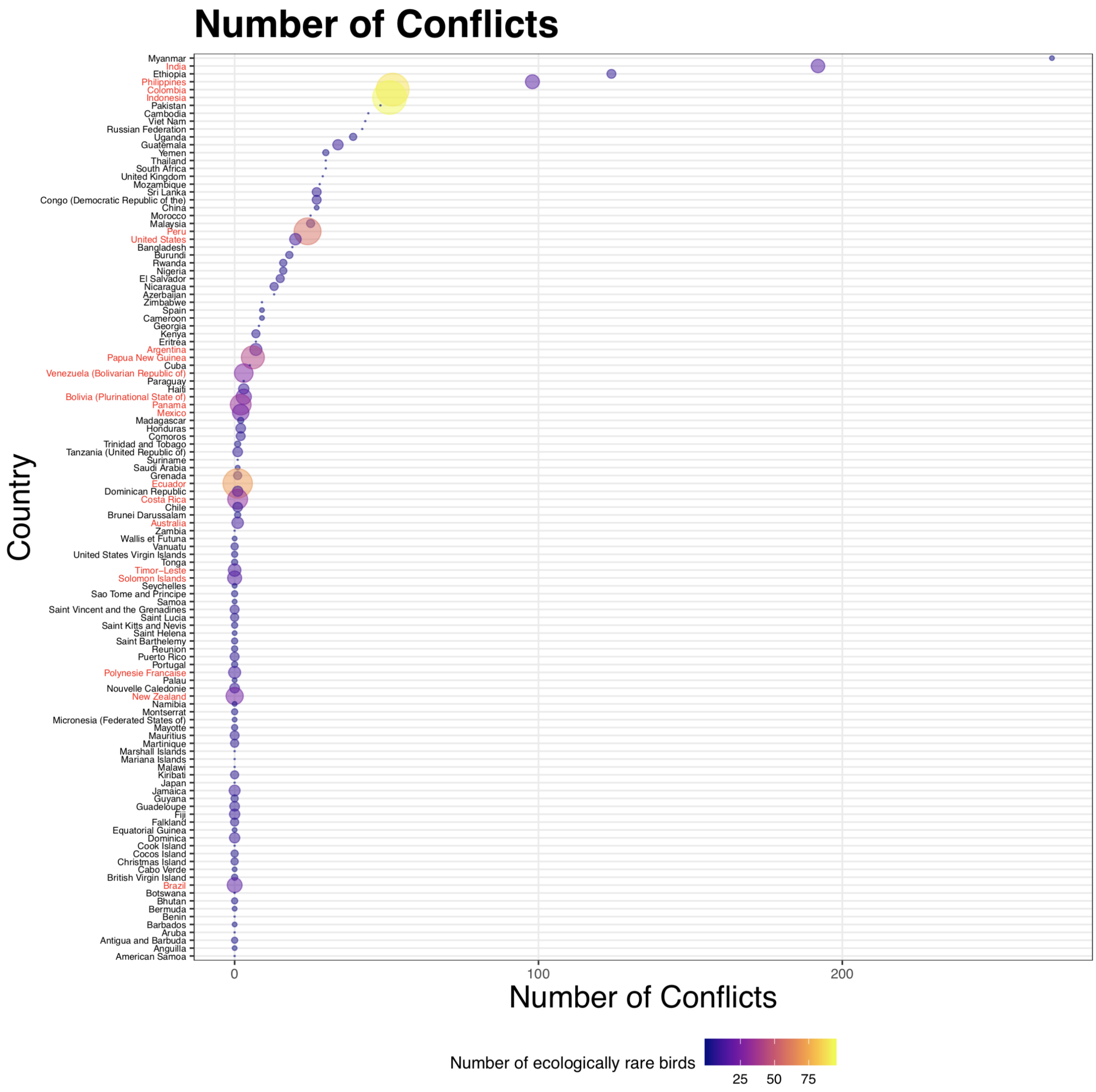

Supplementary Figure 10: HDI (top) and number of conflicts (bottom) in countries hosting ecologically rare birds (118 countries). In red we highlight countries with more than 10 ecologically rare species.

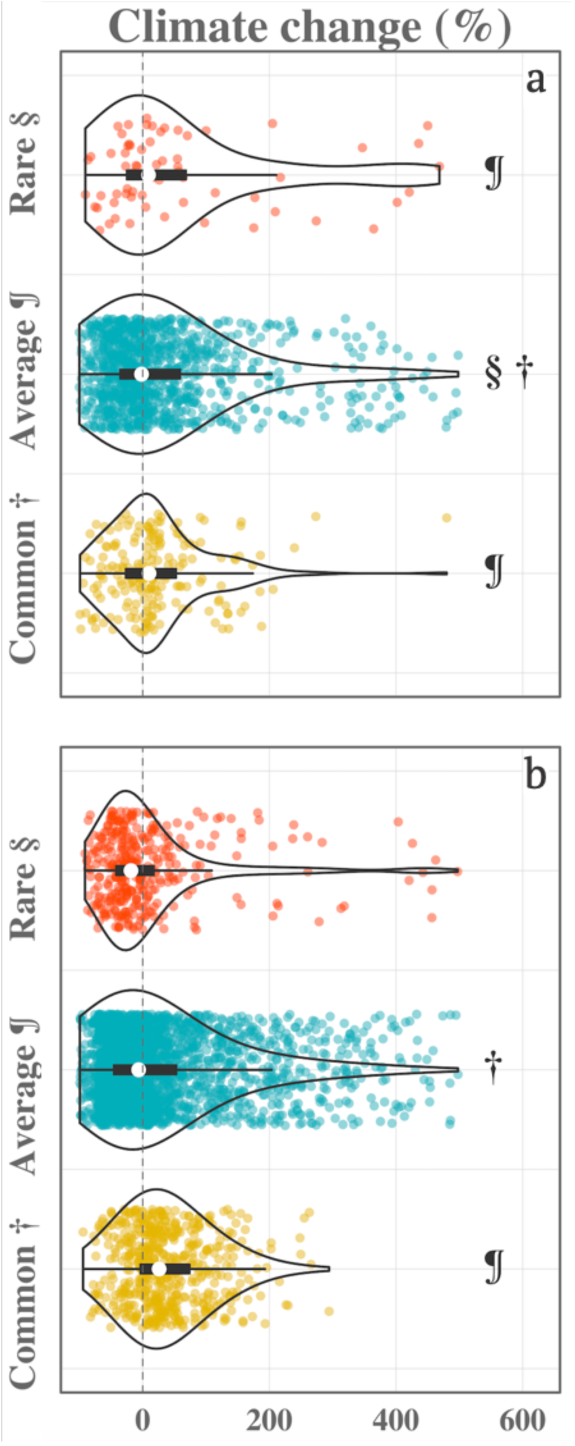

Supplementary Figure 11. Change in distribution range based on climate change projections (scenario RCP 8.5, Horizon 2061 - 2080). Symbol indicate significant similar distribution between groups ($P < 0.05$, See Supplementary Table 3 for all p-value) via one-way ANOVA and Tukey's post-hoc tests.

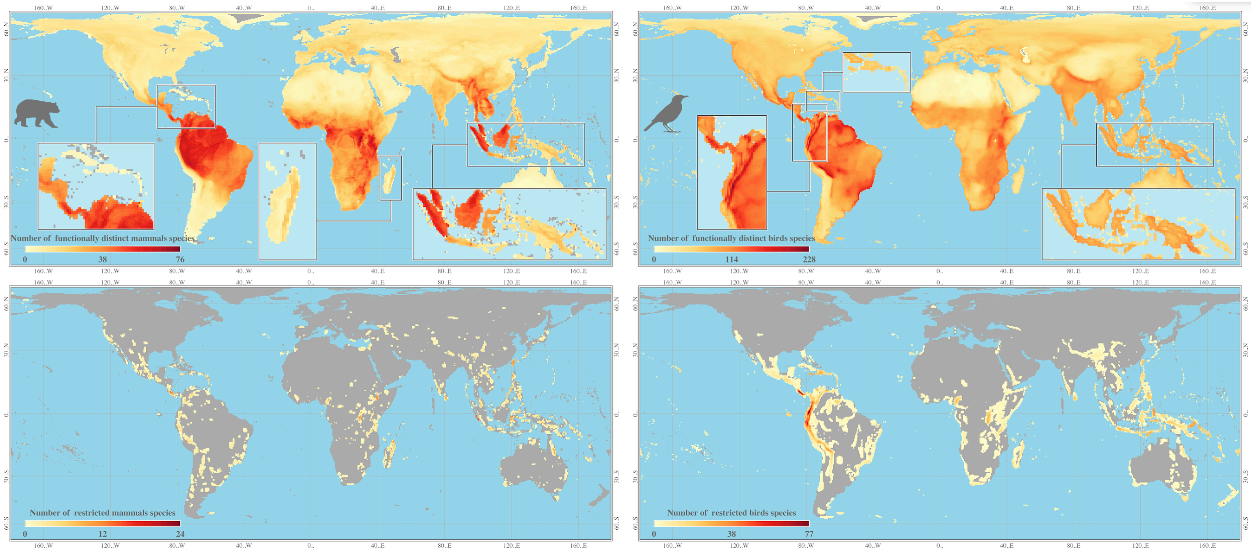

Supplementary Figure 12. Global distribution considering only unglaciated areas of total number of functionally distinct species (first row) and number of geographically restricted species (second row) for mammals (left) and birds (right) species.

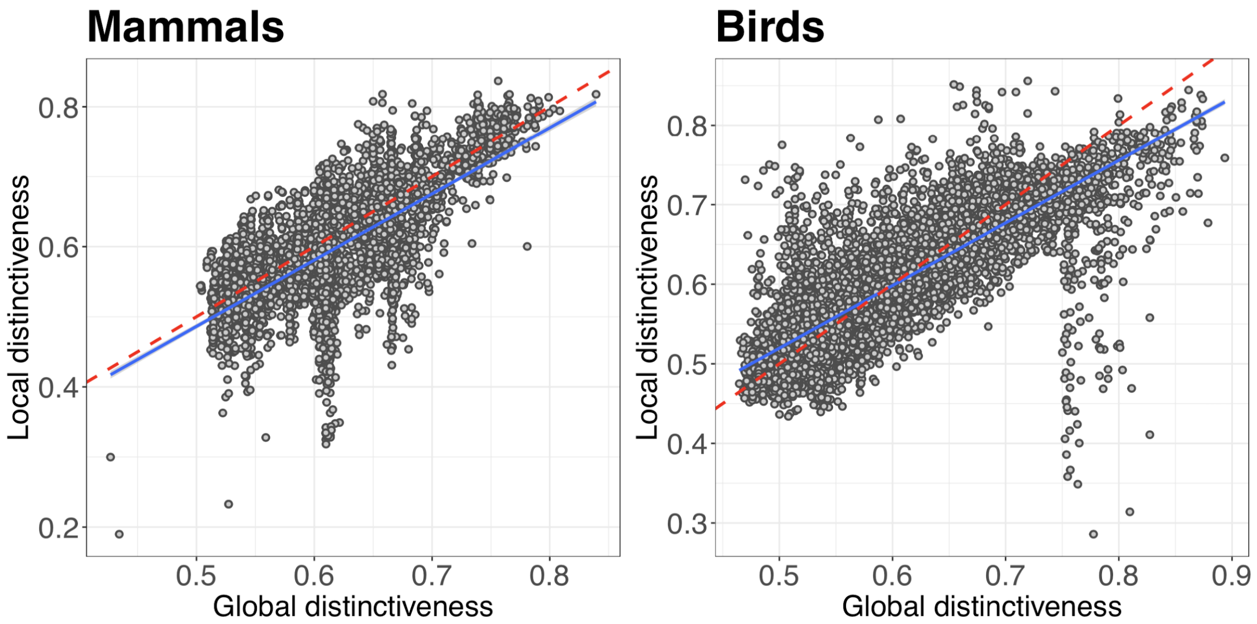

Supplementary Figure 13. Relation between functional distinctiveness computed at global scale and computed at local scale (within cells) for mammals and birds. In blue: linear model between local and global distinctiveness ($R^2 = 0.50$ for mammals, $R^2 = 0.64$ for birds and *p-values* < 0.001). Red lines represent the hypothetical case where global functional distinctiveness is equal to local functional distinctiveness (i.e. first bisector).

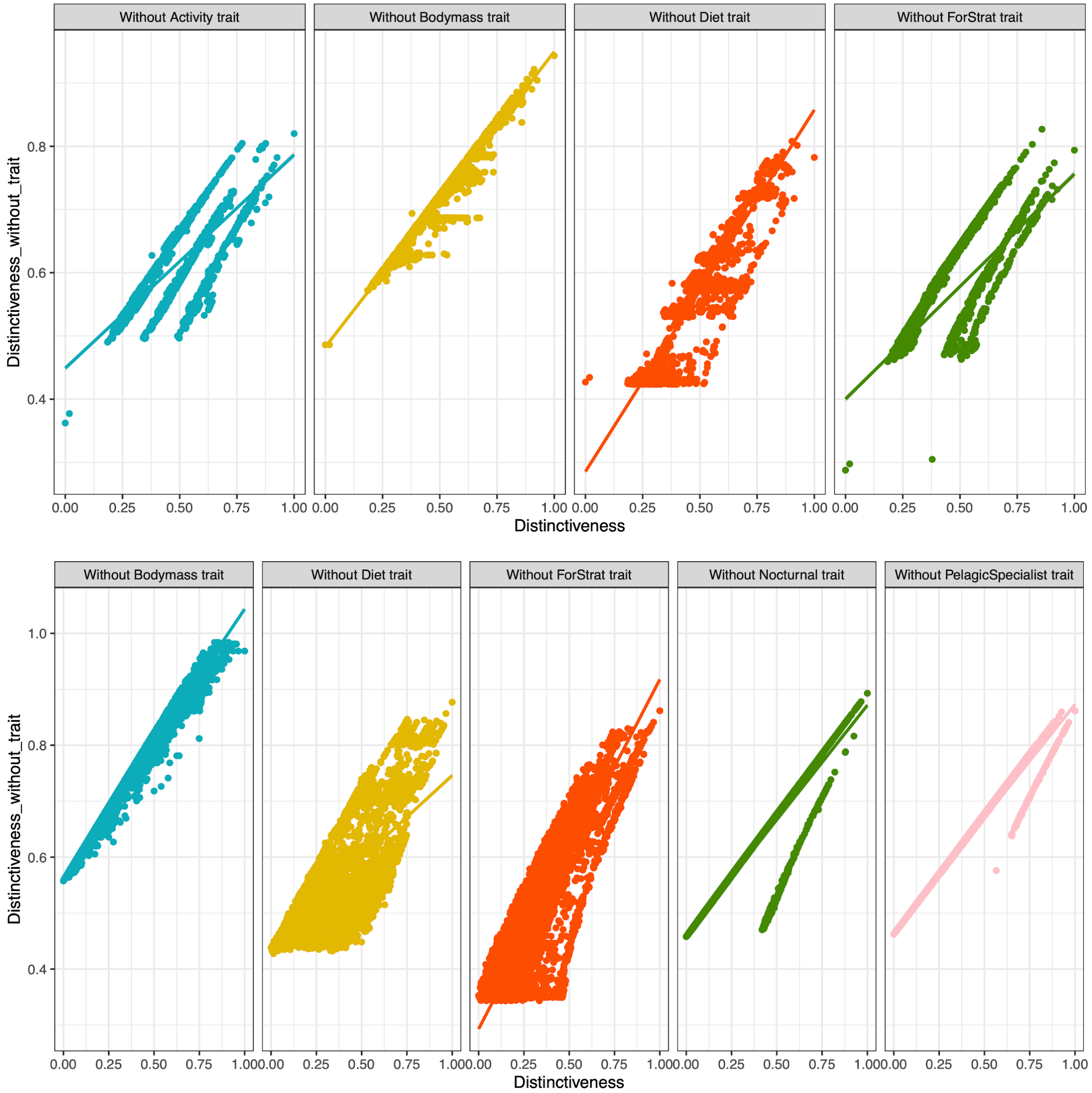

Supplementary Figure 14. To test the sensitivity of distinctiveness to trait choice, each trait was deleted one at a time and distinctiveness was recomputed, then we checked for correlation between initial distinctiveness and distinctiveness with deleted trait. The first row is mammals and second row is birds.

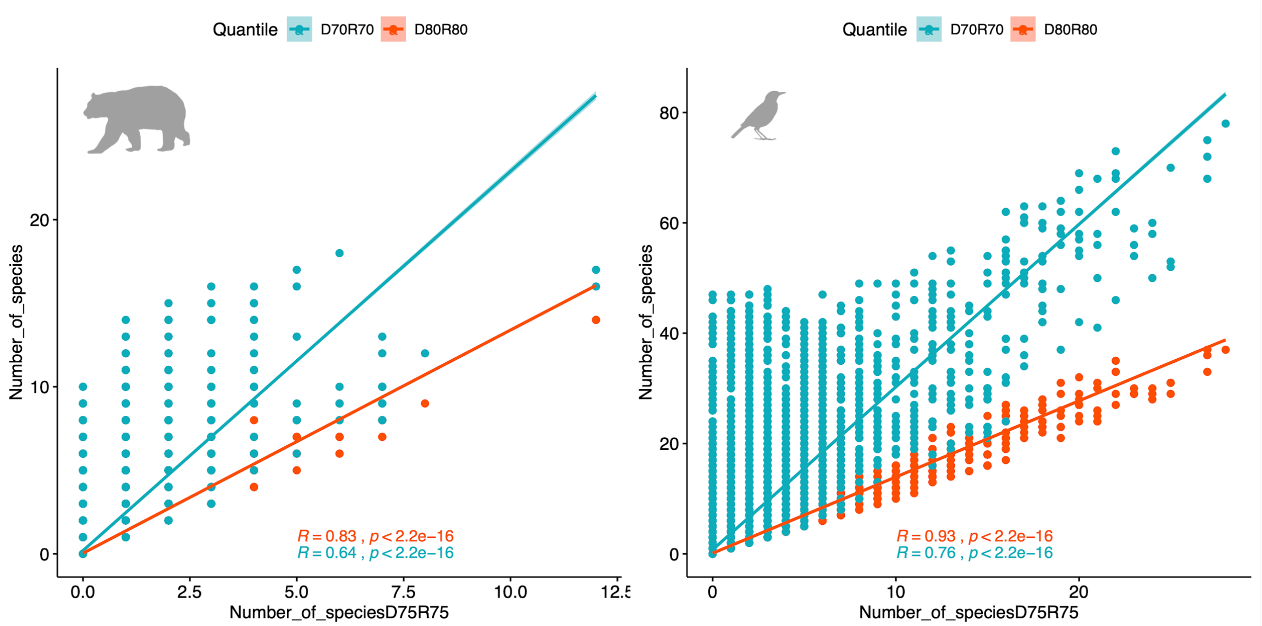

Supplementary Figure 15. We defined *ecologically rare* and *ecologically common* species as having values of functional distinctiveness and geographical restrictiveness either higher than 75% or lower than 25% of the entire species pool of interest. *Ecologically average* species have values of functional distinctiveness and geographical restrictiveness respectively, lower than 75% and higher than 25%. With this approach we reach more or less the 5% of ecologically rare species threshold. We performed a sensitivity analysis to test the influence of the choice of these two thresholds. We defined *ecologically rare* and *ecologically common* species as having values of functional distinctiveness and geographical restrictiveness either higher than 70% and 80%. Then, we test the correlation between the number of ecology rare species defined with the 75% (here D75R75) and these two thresholds 70%(D70R70) and 80%(D80R80). Results show that the spatial distribution of ecological rarity is robust to the choice of the distinctiveness and restrictiveness thresholds to define ecological rare species.

Global biodiversity scenarios

Note that we used Thuiller et al. 2019 framework.

Climatic data

Current climate (1979–2013) was represented by four bioclimatic variables from the CHELSA dataset[1] up-scaled from a 1 km to a 100 km resolution. The chosen variables were as follows: annual mean temperature, annual temperature range, annual sum of precipitation and precipitation seasonality (coefficient of variation in monthly sum of precipitations).Projected future climate variables were taken from five GCMs driven by fours cenarios of RCPs in a factorial manner. The five selected models originate from the CMIP5 collection of model runs used in IPCC's 5th Assessment Report (IPCC 2013). The five models from which data were taken are: CESM1-BGC[2] run by National Center for Atmospheric Research (NCAR); CMCC-CMS[3] run by the Centro Euro-Mediterraneo per i CambiamentiClimatici (CMCC); CM5A-LR[4] run by the Institut Pierre-Simon Laplace (IPSL); MIROC5[5] run by the university of Tokyo; and ESM-MR[6] run by Max PlanckInstitute for Meteorology (MPI-M). Future climatic conditions of the four climatic variables were also taken from the CHELSA dataset[1], which provides CMIP5 scenarios at a native resolution of 30 arc seconds. Future conditions from coarser resolution GCMs had been achieved using climatologically aided interpolation. We took the difference between selected GCMs from CMIP5 at a 0.25° grid cell size for current conditions (1979–2013) and the selected future periods (2041–2060, 2061–2080) and interpolated them using b-spline interpolation to the resolution of 30 arc seconds of CHELSA. The resulting difference was then added to (for temperature) or multiplied with (for precipitation) to the CHELSA climatologies of the 1979–2013 baseline period. As our study used 50 km grid cells, the native CHELSA resolution was upscaled by calculating mean within values per each 50 km grid cell.

Species distribution models

An ensemble of projections of SDM was obtained for all species. The ensemble included projections with Generalized Additive Models, Boosting Regression Trees, Generalized Linear Models and Random Forests. Models were calibrated for the baseline period using 70% of observations randomly sampled from the initial data and evaluated against the remaining 30% data using the true skill statistic (TSS[7]). Presence data were randomly drawn from the gridded range

maps. For absences, we considered data in a reasonable buffer around the presence data to avoid having over-optimistic predictive accuracies[8]. To be consistent with assumed realistic dispersal distances (see next paragraph) and in line with previous analyses, we selected absence data in 3000 and 4000 km buffers around mammal and bird species ranges, respectively. This analysis was repeated four times, thus providing a fourfold internal cross-validation of the models (biomod package[9]). Only models with a TSS > 0.8 were kept and projected into future conditions. For each species, all calibrated models (4 SDMs × 4 repetitions) were then used to project the potential distribution of each species under both current and projected future climatic conditions.

Dispersal limitation

Since most species have a sub-global distribution, we adjusted the area from which species are modelled and for which projections are made. In other words, mammal and birds species, the modelled and projected area included all grid cells within 3000 and 4000 km of species' current distributions, respectively. This represents a maximal dispersal distance and excludes regions and climatic conditions that are outside of what is conceivably within reach for these species[8]. These estimates likely underestimate the true dispersal limitation of most species but give a more reliable estimate than assuming unlimited dispersal during this century.

References

1 Karger, D. N. *et al.* Climatologies at high resolution for the earth's land surface areas. *Scientific data* **4**, 170122 (2017).

2 Lindsay, K. *et al.* Preindustrial-control and twentieth-century carbon cycle experiments with the Earth System Model CESM1 (BGC). *Journal of Climate* **27**, 8981-9005 (2014).

3 Scoccimarro, E. *et al.* Effects of tropical cyclones on ocean heat transport in a high-resolution coupled general circulation model. *Journal of Climate* **24**, 4368-4384 (2011).

4 Persechino, A., Mignot, J., Swingedouw, D., Labetoulle, S. & Guilyardi, E. Decadal predictability of the Atlantic meridional overturning circulation and climate in the IPSL-CM5A-LR model. *Climate dynamics* **40**, 2359-2380 (2013).

5 Watanabe, M. *et al.* Improved climate simulation by MIROC5: mean states, variability, and climate sensitivity. *Journal of Climate* **23**, 6312-6335 (2010).

6 Giorgetta, M. A. *et al.* Climate and carbon cycle changes from 1850 to 2100 in MPI-ESM simulations for the Coupled Model Intercomparison Project phase 5. *Journal of Advances in Modeling Earth Systems* **5**, 572-597 (2013).

7 Allouche, O., Tsoar, A. & Kadmon, R. Assessing the accuracy of species distribution models: prevalence, kappa and the true skill statistic (TSS). *Journal of applied ecology* **43**, 1223-1232 (2006).

8 Barbet-Massin, M. & Jetz, W. The effect of range changes on the functional turnover, structure and diversity of bird assemblages under future climate scenarios. *Global Change Biology* **21**, 2917-2928 (2015).

9 Thuiller, W., Guéguen, M., Renaud, J., Karger, D. N. & Zimmermann, N. E. Uncertainty in ensembles of global biodiversity scenarios. *Nature communications* **10**, 1446 (2019).
