# Supplementary material for: Global distribution and conservation status of ecologically rare mammal and bird species
Source: Nat Commun. 2020 Oct 8;11:5071. doi: 10.1038/s41467-020-18779-w (PMC7545165; doi:10.1038/s41467-020-18779-w)
Supplement: Supplementary file 3 — Reporting Summary [file 41467_2020_18779_MOESM3_ESM.pdf]

nature research

Corresponding author(s): Loiseau Nicolas

Last updated by author(s): 2020/08/31

Reporting Summary

Nature Research wishes to improve the reproducibility of the work that we publish. This form provides structure for consistency and transparency in reporting. For further information on Nature Research policies, see Authors & Referees and the Editorial Policy Checklist .

Statistics

For all statistical analyses, confirm that the following items are present in the figure legend, table legend, main text, or Methods section.

n/a	Confirmed	
☐	☒	The exact sample size (*n*) for each experimental group/condition, given as a discrete number and unit of measurement
☐	☒	A statement on whether measurements were taken from distinct samples or whether the same sample was measured repeatedly
☐	☒	The statistical test(s) used AND whether they are one- or two-sided<br>*Only common tests should be described solely by name; describe more complex techniques in the Methods section.*
☐	☒	A description of all covariates tested
☐	☒	A description of any assumptions or corrections, such as tests of normality and adjustment for multiple comparisons
☐	☒	A full description of the statistical parameters including central tendency (e.g. means) or other basic estimates (e.g. regression coefficient) AND variation (e.g. standard deviation) or associated estimates of uncertainty (e.g. confidence intervals)
☐	☒	For null hypothesis testing, the test statistic (e.g. *F*, *t*, *r*) with confidence intervals, effect sizes, degrees of freedom and *P* value noted<br>*Give P values as exact values whenever suitable.*
☒	☐	For Bayesian analysis, information on the choice of priors and Markov chain Monte Carlo settings
☐	☒	For hierarchical and complex designs, identification of the appropriate level for tests and full reporting of outcomes
☒	☐	Estimates of effect sizes (e.g. Cohen's *d*, Pearson's *r*), indicating how they were calculated

Our web collection on statistics for biologists contains articles on many of the points above.

Software and code

Policy information about availability of computer code

Data collection	No software was used for data collection
Data analysis	All analyses were done using R v.3.6.0 (specific functions within specific package are indicated in italic in the text). All relevant R code is available from the GitHub Repository: https://github.com/FRBCesab/ecorar<br>R code to perform Global biodiversity scenario is available from the GitHub Repository: https://github.com/FRBCesab/free-sdm

For manuscripts utilizing custom algorithms or software that are central to the research but not yet described in published literature, software must be made available to editors/reviewers. We strongly encourage code deposition in a community repository (e.g. GitHub). See the Nature Research guidelines for submitting code & software for further information.

Data

Policy information about availability of data

All manuscripts must include a data availability statement. This statement should provide the following information, where applicable:

- Accession codes, unique identifiers, or web links for publicly available datasets
- A list of figures that have associated raw data
- A description of any restrictions on data availability

All data used in this paper are freely available and downloadable from the web. Species distribution maps were provided by the Mammal Red List Assessment (http://www.iucnredlist.org/). For birds, breeding range distribution maps were extracted from BirdLife (http://www.birdlife.org/). All climatic data are available on the CHELSA data portal (http://chelsa-climate.org). IUCN status are available on the IUCN red list (https://www.iucnredlist.org/). Spatial polygons of protected areas were provided by the World Database on Protected Areas (WDPA, https://www.protectedplanet.net/). We provide for each species, coordinates on the PcoA, value of distinctiveness, restrictiveness and all values of threats analyzed in the present paper (https://github.com/FRBCesab/ecorar).

Field-specific reporting

Please select the one below that is the best fit for your research. If you are not sure, read the appropriate sections before making your selection.

☐ Life sciences ☐ Behavioural & social sciences ☒ Ecological, evolutionary & environmental sciences

For a reference copy of the document with all sections, see nature.com/documents/nr-reporting-summary-flat.pdf

Ecological, evolutionary & environmental sciences study design

All studies must disclose on these points even when the disclosure is negative.

Study description

Here, we characterized the ecological rarity of mammals (4,654 species) and birds (9,287 species). We used the IUCN range maps for 4,787 terrestrial mammal species and the BirdLife range maps for 9,993 bird species. We removed extinct (EX) and extinct in the wild (EW) species. Given that trait and/or phylogenetic information were not available for all species, we only included species for which we had phylogenetic, functional and distribution information (9,287 bird and 4,654 mammal species). Ranges were converted to 50x50 km equal-area grid cells. Mainland cells with > 70% water were excluded but all oceanic island cells with smaller land areas were kept.

We defined ecologically rare and common species as having values of functional distinctiveness and geographical restrictiveness higher than 75% or lower than 25% of the entire species pool.

Using that combined information, we investigated (a) who are the ecologically rare species and how they are distributed across functional space and the tree of life?; (b) how are they spatially distributed and do they follow general biodiversity patterns?; and finally, (c) what current and future threats are they facing in the Anthropocene and are they covered by current protection efforts?

We found that ecologically rare species differ from more common species in both diet and body mass, are insufficiently covered by the current system of protected areas, and are disproportionately sensitive to current and future threats. Combined, these results open the debate on whether conservation priority should be given to ecological rarity in lieu of more widespread and functionally redundant species given future environmental conditions and their uncertainty.

Research sample

We used the IUCN range maps and the BirdLife range maps datasets

IUCN, S. IUCN SSC Guiding Principles on Creating Proxies of Extinct Species for Conservation Benefit. Gland, Switzerland. https://portals. iucn. org/library/sites/library/files/documents/Rep-2016-009. pdf (2016).

BirdLife. BirdLife international. URL, http://www.birdlife.org (2019).

Climate variables describing current climate (1979-2013) derived from the CHELSA dataset (https://chelsa-climate.org/): annual mean temperature, annual temperature range, annual sum of precipitation and precipitation seasonality.

Sampling strategy

Ranges were converted to 50x50 km equal-area grid cells.

Data collection

All data used in this paper are freely available and downloadable from the web. Species distribution maps were provided by the Mammal Red List Assessment (http://www.iucnredlist.org/). For birds, breeding range distribution maps were extracted from BirdLife (http://www.birdlife.org/). All climatic data are available on the CHELSA data portal (http://chelsa-climate.org). IUCN status are available on the IUCN red list (https://www.iucnredlist.org/). Spatial polygons of protected areas were provided by the World Database on Protected Areas (WDPA, https://www.protectedplanet.net/). Human development index was provided by UNDP, Human Development Indices and Indicators: 2018 Statistical Update. (2018). (http://hdr.undp.org/en/2018-update) and Number of armed conflicts by Armed Conflict Dataset (2016). https://ucdp.uu.se/downloads/index.html#armedconflict.

Functional traits for each species (extracted from Wilman et al. 2014)

Phylogenetic tree of mammals and birds (from Jetz et al. 2012 and from Bininda-Emonds et al. 2007)

Please note that all references and websites are provided in our manuscript.

We provide for each species, coordinates on the PcoA, value of distinctiveness, restrictiveness and all values of threats analyzed in the present paper (https://github.com/FRBCesab/ecorar).

Timing and spatial scale

We used the most upgraded datasets provided by IUCN range maps and BirdsLife range maps: Current global distribution range of mammals and birds. Data last updated : 9th May 2020

Data exclusions

We removed extinct (EX) and extinct in the wild (EW) species. Given that trait and/or phylogenetic information were not available for all species, we only included species for which we had phylogenetic, functional and distribution information (9,287 bird and 4,654 mammal species).

Reproducibility

All analyses can be repeated.

Randomization

Not relevant to our study. We defined ecologically rare and common species as having values of functional distinctiveness and geographical restrictiveness higher than 75% or lower than 25% of the entire species pool. However, we performed analyses for all species regardless their level of rarity.

Blinding	Complete blinding

Did the study involve field work? ☐ Yes ☒ No

Reporting for specific materials, systems and methods

We require information from authors about some types of materials, experimental systems and methods used in many studies. Here, indicate whether each material, system or method listed is relevant to your study. If you are not sure if a list item applies to your research, read the appropriate section before selecting a response.

Materials & experimental systems

n/a	Involved in the study
☒	☐ Antibodies
☒	☐ Eukaryotic cell lines
☒	☐ Palaeontology
☒	☐ Animals and other organisms
☒	☐ Human research participants
☒	☐ Clinical data

Methods

n/a	Involved in the study
☒	☐ ChIP-seq
☒	☐ Flow cytometry
☒	☐ MRI-based neuroimaging
